# Lkb1 deficiency confers glutamine dependency in polycystic kidney disease

**DOI:** 10.1038/s41467-018-03036-y

**Published:** 2018-02-26

**Authors:** Ebony M. Flowers, Jessica Sudderth, Lauren Zacharias, Glenda Mernaugh, Roy Zent, Ralph J. DeBerardinis, Thomas J. Carroll

**Affiliations:** 1 Departments of Molecular Biology and Internal Medicine, UT Southwestern Medical Center, Dallas, TX 75390 USA; 2 Children’s Medical Center Research Institute at UTSW, Eugene McDermott Center for Human Growth & Development, Department of Pediatrics, UT Southwestern Medical Center, Dallas, TX 75390 USA; 3 Division of Nephrology and Hypertension, Department of Medicine, Vanderbilt University Medical Center, Nashville, TN 37232 USA; 4 Veteran Affairs Hospital Nashville, Nashville, TN 37232 USA

## Abstract

Polycystic kidney disease (PKD) is a common genetic disorder characterized by the growth of fluid-filled cysts in the kidneys. Several studies reported that the serine-threonine kinase Lkb1 is dysregulated in PKD. Here we show that genetic ablation of Lkb1 in the embryonic ureteric bud has no effects on tubule formation, maintenance, or growth. However, co-ablation of Lkb1 and Tsc1, an mTOR repressor, results in an early developing, aggressive form of PKD. We find that both loss of Lkb1 and loss of Pkd1 render cells dependent on glutamine for growth. Metabolomics analysis suggests that Lkb1 mutant kidneys require glutamine for non-essential amino acid and glutathione metabolism. Inhibition of glutamine metabolism in both Lkb1/Tsc1 and Pkd1 mutant mice significantly reduces cyst progression. Thus, we identify a role for Lkb1 in glutamine metabolism within the kidney epithelia and suggest that drugs targeting glutamine metabolism may help reduce cyst number and/or size in PKD.

## Abstract (web summary)

Polycystic kidney disease (PKD) is characterized by the formation of large fluid-filled cysts. Here Flowers and colleagues show that loss of Lkb1, downregulated in PKD, renders kidney cells dependent on glutamine for growth, and suggest that inhibition of glutamine metabolism may prevent cyst development in PKD.

## Introduction

Polycystic kidney disease (PKD) is one of the most common lethal genetic disorders in humans affecting an estimated 600,000 people in the United States and 7-17 million people worldwide. The disease is characterized by organ overgrowth and the formation of fluid-filled cysts that displace/replace normal renal tissue eventually leading to end-stage renal disease and renal failure. PKD is similar to neoplastic disorders in that the formation and enlargement of cysts is largely due to increased cellular proliferation and growth, abnormal cell polarity, and altered cellular metabolism^1–6^.

The majority of PKD patients have inherited a genetic mutation in one of the polycystin genes, *PKD1* or *PKD2*^7^. Several other genes/pathways involved in kidney development have been implicated in PKD, but the precise cause of cyst formation still remains relatively unclear. Most PKD patients begin to exhibit symptoms between 30 and 50 years of age. However, the age of onset and severity of progression varies greatly and can begin as early as childhood/adolescence. This spectrum of individuals/cases suggests that additional modifications from genetic, epigenetic, or environmental factors contribute to disease pathology and represent a prospective set of therapeutic targets to inhibit or slow cyst progression.

Several classes of drugs have been identified as having potential benefit in autosomal dominant polycystic kidney disease in animal models. However, available treatments, such as Tolvaptan that alleviate cyst enlargement through the regulation of water and sodium transport, have systemic side effects that restrict usage^8,9^. Additional therapies that target cystic/neoplastic tissue while not interfering with normal renal maintenance/function are needed.

Current models suggest that transformed/proliferative tissue undergoes metabolic reprogramming that results in enhanced glycolytic flux^10–14^. A possible explanation for this phenomenon is that glycolysis and coincident activities such as an anaplerotic TCA cycle provide precursors for biomass assimilation in growing cells. Understanding how metabolism is (mis)regulated in PKD may lead to the development of therapeutics that can reduce increased cell proliferation, one of the principal drivers of PKD.

Lkb1/Stk11 is a serine/threonine kinase that was first identified as the causative genetic factor for Peutz−Jeghers syndrome^15,16^ and has since been shown to act as a tumor suppressor in multiple cell types^17^. Lkb1 seems to function in a context-dependent manner that is uncharacteristic of other tumor suppressors. Lkb1 and its primary target, AMP-Kinase, have been shown to regulate energy metabolism in several organ systems^18–21^, and mutations in Lkb1 have been identified in several sporadic epithelial cancers^22^. Although Lkb1 activity has been shown to be abrogated in PKD^6,23^, the precise role that Lkb1 plays in cystogenesis is still uncertain.

Utilizing a combination of in vitro and in vivo techniques, we find that loss of Lkb1 alone is not sufficient to induce cyst formation. However, ablation of Lkb1 enhances cystic growth and accelerates the loss of normal renal parenchyma in kidneys with activated mTOR signaling (Tsc1 mutants). We find that loss of Lkb1 activity renders cells dependent on glutamine for their growth. Similar results are observed in an orthologous model of PKD (Pkd1 mutants). In these kidneys, glutamine is required to maintain pools of nonessential amino acids and glutathione. Excitingly, treatment of mouse models of PKD, including Pkd1 mutants, with drugs that block glutaminolysis mitigates cyst formation in vivo suggesting a potential avenue for PKD therapeutics.

## Results

### Loss of Lkb1 from the collecting ducts does not initiate PKD

**Fig. 1:**
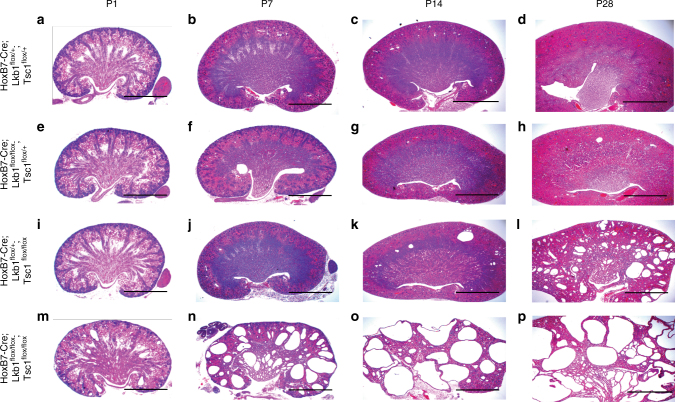
Deletion of Lkb1 enhances cyst progression of Tsc1 mutant kidneys. Representative images of hematoxylin and eosin-stained sections of wild-type (**a**–**d**), Lkb1 mutant (**e**–**h**), Tsc1 mutant (**i**–**l**), and Lkb1/Tsc1 double mutant (**m**–**p**) kidneys at post-natal day 1, 7, 14, and 28. Lkb1 single mutant kidneys displayed no cyst formation. *n* > 5 for each genotype. Scale bars equal 20 mm

Lkb1/Stk11 is a serine/threonine kinase that has been reported to regulate cell proliferation, polarity, mTOR activity, and energy metabolism^6,24–28^, all of which are perturbed in PKD. Furthermore, recent data showed that Lkb1 activity was attenuated in cells lacking *Pkd1*^6,23^, the gene mutated in the majority of cases of human PKD, suggesting that ablation of Lkb1 in the kidney epithelia may be sufficient to cause PKD. A recent study found that deletion of Lkb1 utilizing the Ksp-Cre deleter, which is active throughout the kidney epithelia with highest levels in the distal nephron, led to late-onset tubular atrophy and fibrosis and occasional cyst formation^27^. Somewhat surprisingly, deletion with HoxB7-Cre, a line that is active in the embryonic and adult collecting ducts, had no discernible phenotype upon histological analysis (Fig. 1a–h). To determine if Lkb1 was efficiently deleted, we stained mutant and wild-type kidneys with an antibody to Lkb1. Lkb1 protein levels were significantly decreased in *HoxB7-Cre*; *Lkb1*^*flox/flox*^ collecting ducts at P1 suggesting efficient deletion (Supplementary Fig. 1).

We next sought to determine if any of the processes reported to be regulated by Lkb1 in other contexts were perturbed in mutant collecting ducts without resulting in a significant mutant phenotype. *HoxB7-Cre*; *Lkb1*^*flox/flox*^ and *HoxB7-Cre*; *Lkb1*^*flox/+*^ kidneys were stained with indicators of cell proliferation (proliferating cell nuclear antigen/PCNA), cell death (ACTIVE caspase-3), epithelial polarity (aPKC, ZO-1α), and cilia (acetylated α-tubulin). Lkb1 mutant collecting ducts showed no significant differences in staining pattern of any of these proteins relative to control littermates (Supplementary Figs. 2–4).

### Ablation of Lkb1 enhances cystic growth in Tsc1 mutants

It had previously been suggested that loss of Lkb1 activity in Pkd1 mutant kidney epithelia promoted activation of the mTOR pathway and a switch in cellular metabolism towards glycolysis, a phenomenon referred to as the Warburg effect^23^. The relationship of Lkb1 loss to mTOR pathway status is complicated. There is evidence indicating that mTOR activation in PKD was a direct result of loss of Lkb1 activity^23^, while other reports suggest that activation of mTOR was independent of Lkb1 and that these two pathways cooperated to regulate pAMPK levels^26^. To determine whether the mTOR pathway was perturbed in Lkb1 mutants, wild-type and Lkb1 mutant P1 kidneys were co-stained with cytokeratin, a marker of the collecting duct epithelia, and an antibody specific to the phosphorylated form of S6 ribosomal protein, an indicator of mTORC1 activity (Supplementary Fig. 5). No changes in phospho-S6RP levels were detected in Lkb1 mutant collecting ducts. Thus deletion of Lkb1 alone from the collecting ducts has no effect on mTOR signaling.

One possible explanation for the lack of mTOR activation in Lkb1 mutant collecting ducts is that perhaps Lkb1 does not act upstream or is not the only upstream determinant of this pathway in this cell type. Indeed, Rowe et al. suggested that Lkb1 acted in parallel to the mTOR pathway to cause cyst formation in Pkd1 mutants. To investigate this possibility, we analyzed the effect of Hamartin deletion in the collecting ducts. Hamartin, the protein coded for by Tsc1, cooperates with Tuberin, to form a GAP that represses mTORC1 activity. Tsc1 deletion should be sufficient to activate the mTOR pathway. Indeed, in contrast to the Lkb1 mutants, *HoxB7-Cre; Tsc1*^*flox/flox*^ mice had a late progressing PKD with cysts first appearing between 10 and 14 days of age (Fig. 1i–l). The cysts grew progressively larger as did kidney volume with vast majority of mutants not surviving past 12 months of age. Characterization of the Tsc1 mutant collecting ducts showed that mutant cells were highly proliferative and had a significant increase in mTORC1 activity as indicated by increased levels of phosphorylated S6 ribosomal protein (Supplementary Fig. 5). Thus the mTOR pathway can be activated in the collecting ducts and its activation is sufficient to drive cystogenesis.

To determine whether the Lkb1 and mTOR pathways act in parallel, we generated kidneys with collecting ducts lacking both Lkb1 and Tsc1. Interestingly, ablation of Lkb1 in Tsc1 mutant kidneys greatly accelerated the onset and progression of cystogenesis. Lkb1/Tsc1 double mutant kidneys exhibited cysts as early as embryonic day 15.5 (compared to P14 for Tsc1 mutants and no cysts in Lkb1 mutants; see Fig. 1i–p). Cyst formation progressed rapidly over the next several weeks and by P28, little normal renal parenchyma remained (Fig. 1m–p). The vast majority of *HoxB7-Cre*; *Lkb1*^*flox/flox*^; *Tsc1*^*flox/flox*^ mutants died by 8 months of age.

### Lkb1 mutant embryonic kidneys require glutamine for growth

We next sought to determine how loss of Lkb1 enhanced cystogenesis in Tsc1 mutants. Previous studies reported that Pkd1 mutant cells were glycolytic^5,6,28^, resulting in increased dependence on glucose as a metabolic substrate and that this metabolic switch was at least in part due to loss of Lkb1 activity. We thus hypothesized that Lkb1 deletion altered the metabolic profile of mutant cells, increasing their preference for glucose. To test this, we wished to specifically deprive the kidneys of glucose. As the cystic phenotype was evident in Lkb1/Tsc1 double mutants as early as E15.5, we hypothesized that whatever role Lkb1 was playing, it was first required in the embryonic collecting ducts. Thus, we could use embryonic organ culture as a bioassay to examine metabolite dependence in a modified synthetic lethal screen (synthetic sickness).

To determine if Lkb1 mutant cells were more glucose-dependent than controls, we cultured E12.5 *HoxB7-Cre; Lkb1*^*flox/flox*^ kidneys in glucose-depleted media and assessed growth of the mutant cells. This was accomplished by quantifying UB proliferation rates and branch number. Although Lkb1 mutant ureteric buds grown in the absence of glucose did show defects in their growth rate, there was no significant difference from mutants grown in control (glucose containing) media (Supplementary Fig. 6). In fact, Lkb1 mutants grown in the absence of glucose had higher growth rates than wild-type kidneys grown in the absence of glucose. Thus, Lkb1 deficiency does not enhance glucose dependence, suggesting that alternative nutrients may fuel energy and biosynthetic metabolism in these mutants.

**Fig. 2:**
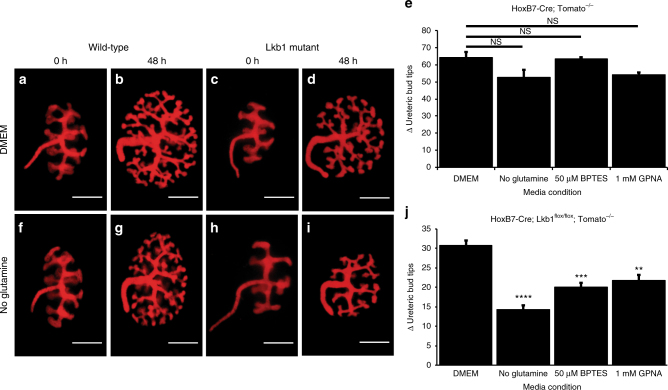
Perturbation of glutamine metabolism causes growth defect in embryonic Lkb1 mutant collecting ducts. Live images of E12.5 HoxB7-Cre; RosaTomato (*n* = 10) (**a**, **b**, **f**, **g**) and HoxB7-Cre; Lkb1^flox/flox^; RosaTomato (*n* = 10) (**c, d, h, i**) kidneys after 0 (**a**, **f**, **c**, **h**) or 48 (**b**, **g**, **d**, **i**) hours of culture in control media (DMEM, **a**−**d**) or DMEM lacking glutamine (**f**–**i**). Quantification of the change (Δ) in branch number (number of ureteric bud tips after 48 h—ureteric bud tips at 0 h) for HoxB7-Cre; RosaTomato (**e**) or HoxB7-Cre; Lkb1^flox/flox^; RosaTomato (**j**) kidneys grown in complete DMEM, DMEM-glutamine, DMEM+ 50 µM concentration of the glutaminase inhibitor BPTES, or 1 mM concentration of the Slc1a5 glutamine transporter inhibitor GPNA. *n* = 10 for each genotype under each condition. Statistical analysis via Mann−Whitney *U*-test **p < 0.01, ***p < 0.001, ****p < 0.0001, NS not significant. Error bars shown as mean ± standard error of the mean (SEM). Scale bars equal 30 microns

Because of its ability to donate both nitrogen and carbon, glutamine serves as a precursor in the generation of amino acids/proteins, nucleotides and lipids and participates in other metabolic processes^29–31^. It has previously been shown that proliferating cells require high levels of glutamine, and that some oncogenic mutations enhance glutamine consumption in a manner similar to the over-utilization of glucose during the Warburg effect^32–35^. To determine if the embryonic collecting ducts were utilizing glutamine as an energetic source, we cultured E12.5 wild-type and Lkb1 mutant kidneys in glutamine-deficient media and quantified UB branching/proliferation. Although wild-type collecting ducts grew normally in the absence of glutamine, Lkb1 mutant kidneys displayed a significant decrease in ureteric bud branching and cell proliferation indicating that loss of Lkb1 resulted in glutamine dependence during cell proliferation (Fig. 2, Supplementary Fig. 7). Previous studies have shown that activation of the mTOR pathway also causes cells to become glutamine dependent. Although our in vivo analysis indicated that loss of Lkb1 did not affect mTOR activity, we tested whether activation of this pathway could also lead to glutamine dependence under these conditions. To test this, we cultured *HoxB7-Cre;Tsc1*^*flox/flox*^ kidneys with and without glutamine. Similar to wild-type cultures, while Tsc1 mutants did require glucose, they did not require glutamine for their growth (Supplementary Fig. 8) in agreement with our previous observation that Lkb1 is not regulating mTOR.

Based on these observations, we hypothesized that the metabolism of glutamine could be targeted as a therapeutic for PKD. Thus we sought to gain insight into the process(es) affected upon glutamine withdrawal.

### Lkb1 mutants require NEAAs and glutathione for growth

Glutamine supplies a variety of metabolic pathways depending on the needs of the cell. In order to substantiate which pathway(s) contributes to collecting duct proliferation within wild-type and Lkb1 mutant kidneys, we used established metabolites and inhibitors of the glutamine/glutamate metabolism pathway in our ex vivo culture system.

We first sought to establish whether glutamine uptake was necessary for branching in the Lkb1 mutant kidneys. To accomplish this we supplemented the culture media with GPNA, an inhibitor of Slc1a5, the primary glutamine transporter, to block glutamine uptake into the cell^29,36^. Inhibition of Slc1a5 resulted in a significant decrease in ureteric bud branching in Lkb1 mutant (but not wild-type) kidneys (Fig. 2), indicating that glutamine uptake by the cell was necessary for growth in Lkb1 mutants.

Once inside the cell, glutamine can be metabolized and utilized in various metabolic processes. To narrow down the requirement for glutamine in Lkb1 mutants, we tested whether inhibition of glutaminase, the enzyme that converts glutamine into glutamate, would lead to a defect in branching in Lkb1 mutants. Similar to what was observed upon withdrawal of glutamine from the media, treatment of Lkb1 mutant kidneys with the glutaminase inhibitor, BPTES^37,38^, resulted in decreased ureteric bud branching relative to wild-type-treated kidneys (Fig. 2), suggesting that glutamine must be metabolized in Lkb1 mutant kidneys to promote growth.

**Fig. 3:**
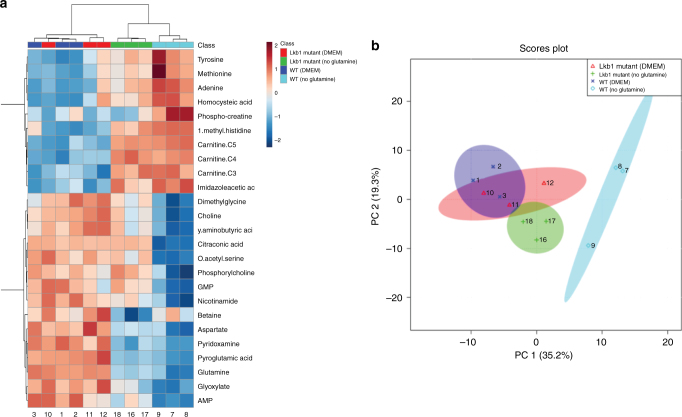
Metabolomics analysis of wild-type and Lkb1 mutant kidneys. Heatmap showing relative abundance of the 25 most significantly affected metabolites extracted from samples of cultured kidneys from embryonic day 13.5 wild-type and Lkb1 mutant kidneys cultured in control media (DMEM) and DMEM-glutamine. The colorbar presented is a log2 scale (**a**). Principal Component Analysis (PCA) of wild-type and Lkb1 mutant kidneys in the presence and absence of glutamine (**b**)

As glutamine derivatives can contribute to multiple biosynthetic processes in the cell, we utilized an unbiased approach to determine which metabolic pathways were deficient in Lkb1 mutants. A metabolomics screen of embryonic wild-type and Lkb1 mutant kidneys in complete media and media lacking glutamine was performed. Surprisingly, these results showed that there was no change in TCA cycle intermediates in Lkb1 mutant kidneys cultured in the absence of glutamine suggesting that the role of Lkb1 loss in cystic enhancement is not due to defects in this pathway. However, we did see significant deficiencies in nucleotide, amino acid, and glutathione metabolism precursors (Fig. 3, Supplementary Fig. 9).

**Fig. 4:**
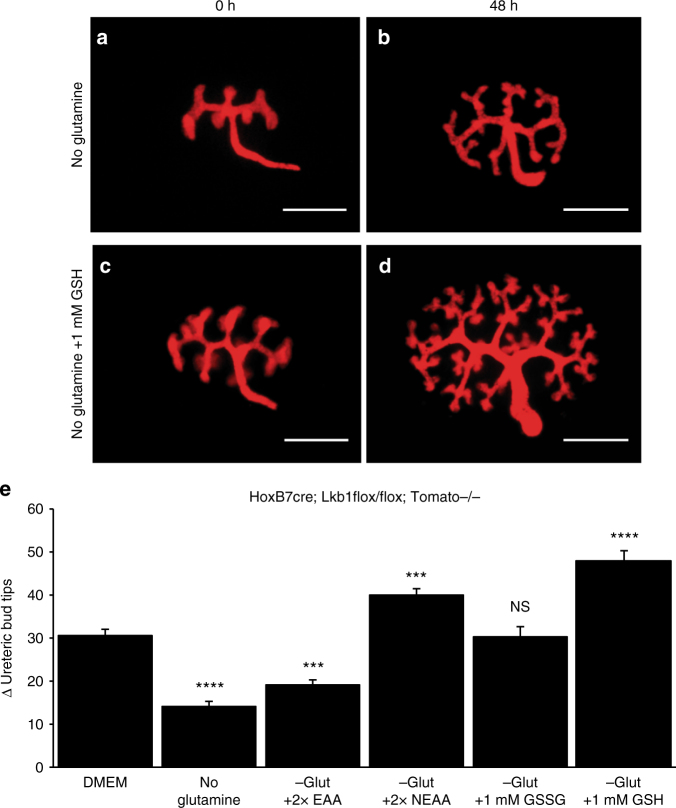
Exogenous sources of amino acids or glutathione rescues growth defect of Lkb1 mutant cells in the absence of glutamine. Live images of e12.5 HoxB7-Cre; Lkb1^flox/flox^; RosaTomato kidneys after 0 (**a**, **c**) or 48 (**b**, **d**) hours of culture in glutamine-deficient media (**a**, **b**) or glutamine-deficient media supplemented with 1 mm GSH (**c**, **d**). **e** Quantification of the change (Δ) in branch number (number of ureteric bud tips after 48 h—ureteric bud tips at 0 h) for e12.5 HoxB7-Cre;Lkb1^flox/flox^; RosaTomato kidneys grown in DMEM, DMEM-glutamine, DMEM-glutamine + 2× essential amino acids, DMEM-glutamine + 2× non-essential amino acids, DMEM-glutamine + 1 mm oxidized glutathione (GSSG), or DMEM-glutamine + 1 mm reduced glutathione (GSH). Note that supplementation with NEAAs, GSSG, or GSH is capable of restoration of Lkb1 mutant collecting duct branching equal to or above levels seen in control media. All statistical comparisons are relative to DMEM conditions. *n* = 10 for each condition. Statistical analysis via Mann−Whitney *U*-test, ***p < 0.001, ****p < 0.0001, NS not significant. Error bars shown as mean ± standard error of the mean (SEM). Scale bars equal 30 microns

To determine whether any of these deficiencies played a causal role in the growth defects observed, we cultured Lkb1 mutant kidneys in glutamine-free media supplemented with essential amino acids, non-essential amino acids (NEAA), or glutathione. A mix of essential amino acids (Sigma) provided minimal but statistically significant enhancement of Lkb1 mutant ureteric bud growth. The addition of oxidized glutathione (GSSG) was capable of rescuing Lkb1 mutant kidneys to control media conditions. However, augmentation with NEAA or reduced glutathione increased ureteric bud branching to levels comparable to what is observed in wild-type kidneys cultured in complete media suggesting that defects in one or both of these biosynthetic pathways may be contributing to the growth defects observed in Lkb1 mutant kidneys (Fig. 4).

### Pkd1 mutants require NEAAs and glutathione for growth

**Fig. 5:**
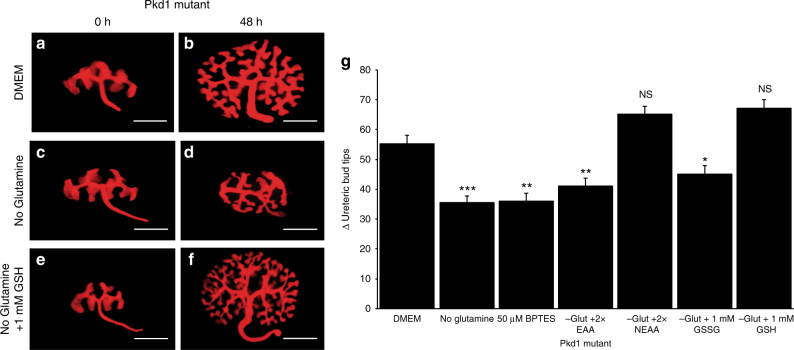
Embryonic Pkd1 mutant collecting ducts are dependent on glutamine for growth. Live images of e12.5 HoxB7-Cre; Pkd1^flox/flox^; RosaTomato kidneys after 0 (**a**, **c**, **e**) or 48 (**b**, **d**, **f**) hours of culture in complete media (DMEM, **a**, **b**), glutamine-deficient media (**c**, **d**) or glutamine-deficient media supplemented with 1 mm GSH (**e**, **f**). **g** Quantification of the change (Δ) in branch number (number of ureteric bud tips after 48 h—ureteric bud tips at 0 h) for E12.5 HoxB7-Cre; Pkd1^flox/flox^; RosaTomato kidneys grown in DMEM, DMEM-glutamine, DMEM + BPTES, DMEM-glutamine + 2× essential amino acids, DMEM-glutamine + 2× non-essential amino acids, DMEM-glutamine + 1 mm oxidized glutathione (GSSG), or DMEM-glutamine + 1 mm reduced glutathione (GSH). Statistical analysis is relative to Pkd1 mutant grown in complete media (DMEM). *n* = 10 for each media condition. Statistical analysis via Mann−Whitney *U*-test. *p < 0.05, **p < 0.01, ***p < 0.001, NS not significant. Error bars shown as mean ± standard error of the mean (SEM). Scale bars equals 30 microns

Although previous studies have suggested that Pkd1 mutants have decreased Lkb1 activity^6,23^, a similar glutamine dependence has not been shown. To determine If Pkd1 mutants also show a dependence on glutamine, E12.5 *HoxB7-Cre; Pkd1*^*flox/flox*^ kidneys were cultured for 48 h in complete media or media lacking glutamine and cell proliferation rates and UB branching were quantified. Similar to what was observed in Lkb1 mutants, we observed a significant decrease in Pkd1 mutant ureteric bud branching when glutamine was withdrawn from the culture media suggesting that Pkd1 mutant kidneys also require glutamine for their growth (Fig. 5). To determine if this glutamine dependence required glutaminolysis, we first sought to determine whether glutaminase activity was required. Similar to what was observed with Lkb1 mutants, addition of BPTES to the culture media inhibited growth in Pkd1 mutants but not in wild-type cultured kidneys. Further, analogous to what we observed in Lkb1 mutant kidneys, an exogenous source of essential amino acids or oxidized glutathione (GSSG) was sufficient to increase proliferation and branching compared to glutamine withdrawal, but neither was capable of increasing growth to control media conditions. In contrast, the supplementation of NEAA or reduced glutathione (GSH) to glutamine-free media was capable of returning Pkd1 mutant kidney ureteric bud growth to control levels (Fig. 5).

### Inhibition of glutaminase represses cyst progression in vivo

**Fig. 6:**
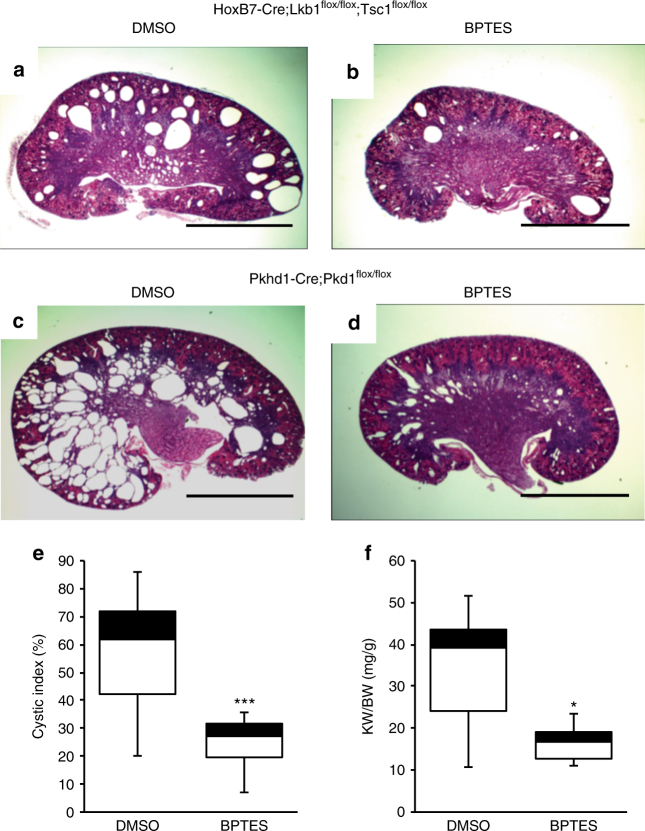
Inhibition of glutamine metabolism slows cyst progression in vivo. Representative examples of H&E-stained sections from post-natal day 10 HoxB7-Cre; Lkb1^floxflox^; Tsc1^flox/flox^ (**a**, **b**); and Pkhd1-Cre; Pkd1^flox/flox^ (**c**, **d**) kidneys that were treated with 5% DMSO (**a**, **c**) or 100 mg kg^-1^ body weight of the glutaminase inhibitor, BPTES (**b**, **d**). Cystic indices for DMSO vs. BPTES-treated Pkhd1-Cre; Pkd1^flox/flox^ kidneys show a significant reduction in the number of cysts in BPTES-treated animals (**e**). Kidney-to-body weight ratio of DMSO vs. BPTES-treated Pkhd1-Cre; Pkd1^flox/flox^ kidneys (**f**). HoxB7-Cre; Lkb1^flox/flox^; Tsc1^flox/flox^ (DMSO-treated, *n* = 16; BPTES-treated, *n* = 16). Pkhd1-Cre; Pkd1^flox/flox^ (DMSO-treated, *n* = 14; BPTES-treated, *n* = 14). Statistical analysis via Mann−Whitney *U*-test. *p < 0.05, ***p < 0.0001. Error bars shown as mean ± standard error of the mean (SEM). Scale bars equals 20 mm

Our data suggest that glutaminolysis is necessary for proliferation in Lkb1 and Pkd1 mutant embryonic kidneys. As Lkb1 activity appears to be lost in Pkd1 mutants and as Lkb1 can suppress cystic growth in adult kidneys, we hypothesized that we may be able to target pathological growth in cystic kidneys in vivo by targeting glutamine metabolism. To test this, we administered the glutaminase inhibitor, BPTES (100 mg kg^−1^), to pregnant females carrying litters of *HoxB7-Cre; Lkb1*^*flox/flox*^; *Tsc1*^*flox/flox*^ embryos beginning at E15.5. Unfortunately, the administration during embryogenesis lead to spontaneous termination of the pregnancy. To overcome this issue, we administered the drug to nursing mothers from post-natal day 1 to post-natal day 10. Kidneys were harvested from *HoxB7-Cre; Lkb1*^*flox/flox*^; *Tsc1*^*flox/flox*^ mutants and subsequently sectioned, stained and their cystic burden quantified. We observed a significant decrease in cystic index in mutant pups whose mothers were administered BPTES vs. ones whose mothers were administered the vehicle alone (Fig. 6a, b, Supplementary Fig. 10a).

Although our data suggest that the Lkb1/Tsc1 mutants mimic the etiology of human PKD, we wanted to test the therapeutic potential of glutaminase inhibitors in an orthologous model of the disease. To accomplish this, we generated mice in which Pkd1 was deleted with Pkhd1-Cre. Previous analysis showed that this Cre line is primarily active in the postnatal collecting ducts^39^ allowing us to determine whether the glutamine dependence was only relevant to embryonic stages or also in a post-natal state.

*Pkhd1-Cre; Pkd1*^*flox/flox*^ pups were administered glutaminase inhibitor through the breast milk and harvested 10 days later. Similar to what was observed with the Lkb1/Tsc1 double mutants, administration of the glutaminase inhibitor significantly decreased the kidney-to-body weight ratio and cystic burden in *Pkhd1-Cre*; *Pkd1*^*flox/flox*^ pups (Fig. 6c, d). Although serum creatinine levels become elevated in older mutants, we were unable to detect significant differences in creatinine levels between untreated wild-type and mutant mice (treated or untreated) at P10 (Supplementary Fig. 10b).

### Glutamine is essential for growth of adult Lkb1 mutant cells

To this point, our studies have focused on embryonic and early post-natal kidneys. However, the majority of human PKD patients do not develop cysts until 30−50 years of age. To determine if our findings would be relevant to the adult kidney, we generated cell lines from the collecting duct of 6-to-8-week-old *Lkb1*^*flox/flox*^ and *Pkd1*^*flox/flox*^ mice. Individual lines were transfected with adenoviruses expressing ubiquitous Cre to generate mutant cell lines.

In contrast to what we observed with our intact embryonic kidney cultures, control, Lkb1 mutant, and Pkd1 mutant cells all died within 48 h if glutamine was completely withdrawn from the media (Supplementary Fig. 11a). This is in agreement with previous studies showing that SV-40 immortalization alone was sufficient to cause glutamine addiction. To determine if mutant cells were more sensitive to glutamine deprivation, we cultured cells in different concentrations of glutamine. Similar to what was observed in our ex vivo models, isolated Lkb1 mutant collecting duct cells were more dependent on glutamine than control cells, and this dependence led to a significant decrease in proliferation in a concentration-dependent manner (Supplementary Fig. 11b). The growth defect of Lkb1 mutant cells in glutamine-deprived conditions could be rescued by the addition of an exogenous source of NEAA (-glutamine), as well as reduced glutathione (Supplementary Fig. 11b, c). Addition of nucleotides/nucleosides (Millipore) to glutamine-depleted media was unable to rescue the proliferation defect of Lkb1 mutants (Supplementary Fig. 11c). However, isolated adult Pkd1 mutant cells did not respond in a similar manner to glutamine withdrawal as Pkd1 embryonic cultures. Pkd1 mutant cells had no significant decrease in proliferation compared to control cells under low glutamine conditions. Further, while the addition of NEAA or reduced glutathione to low glutamine media (0.25 mm) rescued the Lkb1 mutants, it actually caused a significant decrease in proliferation of both wild-type and Pkd1 mutant cells (Supplementary Fig. 11b, d). These data suggest that while loss of Lkb1 renders embryonic and adult collecting duct cells glutamine dependent, loss of Pkd1 results only in glutamine dependence in the embryonic/earl post-natal stage but not in adults. This may explain why deletion of Pkd1 from adult kidneys leads to a very slow progressing cystic disease.

## Discussion

There have been many attempts to therapeutically target increased cell proliferation in human PKD, although to date, no therapeutics have been approved for human use in the US. Here, we show that embryonic kidneys lacking Pkd1 require glutamine for cell proliferation and tissue growth. Our data suggest that this is at least in part due to loss of Lkb1 activity as Lkb1 mutants show a similar glutamine dependence and co-ablation of Lkb1 enhances cyst formation in Tsc1 mutants. Importantly, ablation of Tsc1 alone does not lead to glutamine dependence (not shown). Inhibition of glutaminolysis both in ex vivo organ culture and in vivo slows cell growth and blocks cyst progression. Thus, we have revealed a novel metabolic pathway that can be targeted for PKD therapy.

We acknowledge that PKD is not solely caused by increased proliferation^40^ and defects in solute transport most likely contribute to the disease. However, therapies that can slow proliferation should have a significant impact on this disease and can potentially be coupled with therapies that regulate tubular secretion. We feel that the data collected in this study will have a direct and significant impact on PKD treatment.

Mechanistically, our data suggests that Lkb1 normally acts downstream of Polycystin1 to suppress cell proliferation within the kidney epithelia. However, it is important to note that our data does not suggest that defects in glutamine metabolism contribute to PKD. This is highlighted by the fact that loss of Lkb1 alone is not sufficient to cause cyst formation. The metabolic alterations themselves are not cyst causing. Instead, our data suggests that loss of Lkb1 activity alters some aspect of normal metabolism rendering mutant cells more dependent on glutamine than wild-type. In other words, glutamine metabolism is an Achilles’ heel in PKD. Although usually considered an NEAA, glutamine is actually a conditionally essential amino acid, meaning under certain conditions, the body is not able to synthesize sufficient amounts to fulfill metabolic requirements. Indeed glutamine appears to be essential for rapid cell growth and many cancers require high rates of synthesis and/or import for their growth/survival^30,32,33,41,42^. This conditional dependence makes targeting glutamine dependence particularly attractive as it should only affect the diseased cells leaving normal, healthy tissue unaffected (as we observed in our ex vivo and in vivo studies).

In other pathological conditions, alterations in various molecular processes have been reported to underlie glutamine dependence. Our metabolomic analysis indicates that in Lkb1 mutants, glutamine is required for NEAA and glutathione synthesis. Ex vivo culture experiments revealed that the defect in growth caused by the absence of glutamine can be completely rescued with an exogenous source of NEAA and reduced glutathione (GSH), while the addition of essential amino acids shows only moderate enhancements in growth, relative to NEAA and GSH.

The changes in the NEAA and glutathione metabolic pathways may be related. Glutathione, an antioxidant that prevents cellular damage from reactive oxygen species (ROS), consists of three NEAA precursors (glutamate, glycine, and cysteine), and the production rates of these amino acids are strictly controlled by cells to optimize glutathione utilization for redox reactions. The ability of the oxidized form of glutathione to rescue at low efficiency may be the result of the cells converting it back to the reduced form. It is possible that the absence of glutamine slows the production of one or more of the required glutathione precursors and increases the vulnerability of mutant cells to energetic stress and ROS. However, it is possible that this has nothing to do with ROS and glutathione is simply able to be metabolized into some other metabolite (such as glutamate and cysteine) that is necessary for growth. Further investigation into what genetic, epigenetic, and environmental alterations occur within mutant cells during glutamine withdrawal is needed to determine how these metabolic shifts cause significant changes in cellular behavior including cell stability, proliferation, and repair. It is still not clear where and how Lkb1 is functioning to regulate metabolism in the collecting ducts. We found that deletion of Lkb1 does not affect the levels of pAMPK in collecting duct cells in vivo or in vitro under otherwise normal conditions; thus the substrate of Lkb1 within the collecting ducts is still not clear. Indeed it is not even certain that this role requires the kinase activity of this protein.

A final important observation that comes from this study is that organ culture can be used as a bioassay for PKD therapeutics. In the past, this was not considered to be the case as Pkd1 and Pkd2 mutant kidneys do not form cysts when cultured ex vivo. Although addition of cAMP agonists lead to tubule dilation, the physiological relevance of this treatment is unclear^2^. Here, we show that growth of the embryonic kidney can be used as a surrogate for pathological growth, and this can be easily visualized by monitoring branching morphogenesis. By performing a modified synthetic lethal screen (synthetic sickness), we were able to identify a crucial role for glutamine in kidney growth, the biological process in which it was acting, and identify potential therapeutics.

Because our studies were performed in embryonic and early post-natal kidneys, it is possible that these findings may not extend to the adult disease. In fact, while deletion of Lkb1 in adult collecting duct cell lines resulted in the same metabolic changes as seen in the embryo/post-natal collecting duct, Pkd1 mutants behaved similar to wild-type in terms of dependence on glutamine and response to NEAA and glutathione. This could be interpreted to mean that Pkd1 deletion in adult kidneys does not lead to glutamine dependence. However, another possibility is that ablation of Pkd1 in adult cells is not sufficient to alter metabolism without some additional stress. Indeed, previous work has shown stark differences in PKD pathology depending on the developmental stage in which Pkd1 is ablated^43^. Ablation of Pkd1 in adult kidneys leads to a very slow progressing disease relative to ablation in embryos/early post-natal animals. The lack of glutamine dependence in adult Pkd1 mutant cells may reflect this developmental switch. However, given that ablation of Lkb1 from the adult does lead to glutamine dependence, this model would predict that adult ablation of Pkd1 does not affect Lkb1 activity. Importantly, Polycystin1 had only been shown to regulate Lkb1 activity in embryonic fibroblasts and early postnatal kidneys^23,44^. To our knowledge, it has not been analyzed in mutant adult kidneys. It will be interesting to determine if Lkb1 activity plays a role in this developmental switch. A second possibility is that Lkb1 and/or glutamine only play a role in immature tubules and they have no role in adult PKD. Studies to address these questions are underway. Interestingly, cells derived from an autosomal recessive form of PKD in rats also show glutamine dependence suggesting that glutamine metabolism may represent a therapeutic target for multiple forms of PKD^45^.

In summary, we have identified a metabolic alteration in PKD caused at least in part to deficits in Lkb1 activity that makes cells sensitive to glutamine withdrawal. We believe that a greater insight into the precise molecular role of Lkb1 and metabolism in cyst progression will advance our understanding of PKD etiology as well as identifying additional therapeutic targets.

## Methods

### Mice

HoxB7-Cre, Pkhd1-Cre, Lkb1^flox/flox^, Tsc1^flox/flox^ and Pkd1^flox/flox^ have been previously described^46–50^. All mice were bred on a mixed genetic background. For each experiment, female mice 7−8 weeks of age were crossed with a male 9−10 weeks of age. For initial histological and immunohistological characterization experiments, 3−4 pregnant Lkb1^flox/flox^, Tsc1^flox/flox^, and Lkb1^flox/flox^Tsc1^flox/flox^ females were crossed with one HoxB7-Cre; Lkb1^flox/+^, HoxB7-Cre; Tsc1^flox/+^, or HoxB7-Cre; Lkb1^flox/+^; Tsc1^flox/+^ male. For ex vivo culture experiments, plugs were checked and embryos were collected at embryonic day 12.5 for further analysis. Mice of both sexes with the desired genotype were randomly selected and the investigator was blinded to allocation. All animals were housed, maintained, and used according to National Institutes of Health (NIH) and Institutional Animal Care and Use Committees (IACUC) approved protocols at the University of Texas Southwestern Medical Center (OLAW Assurance Number D16-00296).

### Mouse genotyping

Genotype sequencing information is given in Supplementary Table 1.

### Histology/immunofluorescence of sectioned kidneys

For histological analysis, post-excised kidneys were fixed in 4% paraformaldehyde, embedded in paraffin, sectioned into 5 µm slices, and subjected to hematoxylin and eosin staining. For immunohistochemistry, post-excised kidneys were fixed with 4% paraformaldehyde, cryoprotected with 30% sucrose, and frozen embedded in OCT medium (TissueTek). 10 µm frozen sections were washed with PBS with 0.1% Triton-X or 1× TBS with 0.1% Tween-20. Slides were immersed and boiled in 10 mm sodium citrate antigen retrieval buffer and blocked with a solution of 5% FBS/TBS-Tween-20 for 1 h at room temperature followed by the application of primary antibodies diluted in blocking solution. The following antibodies were used: aPKC (atypical protein kinase C; Santa Cruz, Cat. sc-216, dilution 1:500), Lkb1 (liver kinase B1; Santa Cruz, Cat. sc-133742, dilution 1:300), PCNA (proliferating cell nuclear antigen; Santa Cruz, Cat. sc-7907, 1:500), ZO-1α (Zonula occludens-1alpha; Santa Cruz, Cat. sc-33725, dilution 1:500), α-tubulin (acetylated alpha-tubulin; Cell Signaling, Cat. 5335, dilution 1:800), pS6RP (phosphorylated-S6 ribosomal protein; Cell Signaling, Cat. 4858, dilution), caspase-3 (ACTIVE^®^ caspase-3; Promega, Cat. G748A, dilution 1:500), CK (pan-cytokeratin; Sigma-Aldrich, Cat. C2562, dilution 1:500). Secondary antibodies were diluted in blocking solution (1:500 AlexaFluor^®^488-conjugated anti-rabbit IgG, 1:500 AlexaFluor^®^568-conjugated anti-mouse IgG) for 1 h at room temperature. Nuclei were counterstained using either Topro-3 (Invitrogen, dilution 1:1000) or DAPI (Sigma, 1:1000), then mounted with 50% glycerol (FisherScientific) in PBS, and sealed with a coverslip.

### Ex vivo whole kidney culture

Organ culture of isolated embryonic day 12.5 (E12.5) metanephric kidneys were performed as previously described [51,52]. Embryonic day 12.5 metanephric kidneys were isolated and cultured on polycarbonate filters (pore-size of 0.8 μm) in a 24-well plate (Corning, Costar). They were cultured on an air-medium interface with media consisting of Dulbecco’s modified Eagle’s medium (DMEM) with 10% fetal bovine serum (Sigma), and 1% antibiotic/antimycotic solution (Corning) with or without glucose and/or glutamine for 2 to 5 days. Bis-2-(5-phenylacetamido-1,3,4-thiadiazol-2-yl)ethyl sulfide (BPTES), gamma-l-glutamyl-p-nitroanilide (GPNA), essential amino acid mix, NEAA mix (without glutamine) oxidized glutathione disodium salt (GSSG) and reduced glutathione (GSH), were all purchased from Sigma-Aldrich and were added to the media at the concentrations listed in the figures. All culture dishes were incubated in a fully humidified 37 °C incubator with 5% CO_2_. Quantitation of the change (Δ) in branching events was measured by counting the ureteric bud tips for each selected image immediately after the culture period began (*T* = 0 h) and after a 48-h time-lapse.

### Generation of cell lines

Collecting duct cells were isolated from the tip of the renal papilla of 4-week-old Lkb1^flox/flox^ or Pkd1^flox/flox^ mice and immortalized by infection with T-antigen^53^. Clonal lines were generated by serial dilution. Null cell lines were generated from floxed lines by infection with adenovirus carrying a ubiquitously expressed Cre transgene. Ablation of the floxed allele was confirmed by genomic PCR. Several independent lines were derived for each genotype.

### Metabolomics

E13.5 HoxB7-Cre;RosaTomato and HoxB7-Cre;Lkb1^flox/flox^; RosaTomato kidneys were harvested, cultured in control media (DMEM) or DMEM lacking glutamine for 24 h. Kidneys were then washed twice with ice-cold saline, dried, transferred to an Eppendorf tube, and flash-frozen with liquid nitrogen. For metabolite purification, extraction, and analysis, kidneys were submerged in 500 μl of cold methanol/water (50/50, v/v), subjected to three freeze-thaw cycles, vortexed, and centrifuged. The supernatant was transferred to a new tube, air-dried, and metabolites were reconstituted for metabolomics analysis via LC/MS (Agilent 6550 iFunnel LC/quadrupole-time of flight mass spectrometer^54^). Samples for each genotype, containing approximately 20 embryonic kidneys per sample, were generated for each media condition before processing. Pellets from each sample were used for protein quantification. Statistical differences were determined via Partial Least Squares-Discriminant Analysis (PLS-DA).

### In vivo treatments

Pregnant or nursing dams were injected intraperitoneally once a day with BPTES (100 mg/kg) or vehicle (5% DMSO in PBS) from E15.5 till birth or beginning on post-natal day 1 until post-natal day 10. Tissues were then harvested from vehicle or BPTES-treated HoxB7-Cre; Lkb1^flox/flox^; Tsc1^flox/flox^ or Pkhd1-cre; Pkd1^flox/flox^ neo-natal mice 12 h after final injection. Post-excised kidneys were fixed with 4% paraformaldehyde overnight, dehydrated with a series of ethanol solutions, sectioned, and stained with hematoxylin and eosin as described above. Cystic indices were conducted by overlaying a grid composed of 0.1 inch^2^ to H&E-stained sagittal kidney sections. Cystic index is presented as the percent of total kidney area that is occupied by cysts. HoxB7-Cre; Lkb1^flox/flox^; Tsc1^flox/flox^ (DMSO-treated, *n* = 16; BPTES-treated, *n* = 16). Pkhd1-Cre; Pkd1^flox/flox^ (DMSO-treated, *n* = 14; BPTES-treated, *n* = 14).

### Statistical analysis

All data presented as the mean ± the standard error of the mean (SEM). Statistical difference between ex vivo organ culture treatments was performed using two-tailed Mann−Whitney *U*-test or Student’s *t* test. *P* value < 0.05 was considered statistically significant for all experiments. For all animal/tissue studies, a minimum of three replicates from at least two different litters were performed.

### Data availability

The authors declare that all primary and supplementary data associated with the results of this study are available within the manuscript or supplementary information. Any additional data are available upon sensible request.

## Electronic supplementary material

Supplementary Information

Peer Review File
