# Supplementary material for: Lkb1 deficiency confers glutamine dependency in polycystic kidney disease
Source: Nat Commun. 2018 Feb 26;9:814. doi: 10.1038/s41467-018-03036-y (PMC5827653; doi:10.1038/s41467-018-03036-y)
Supplement: Supplementary file 1 — Supplementary Information [file 41467_2018_3036_MOESM1_ESM.pdf]

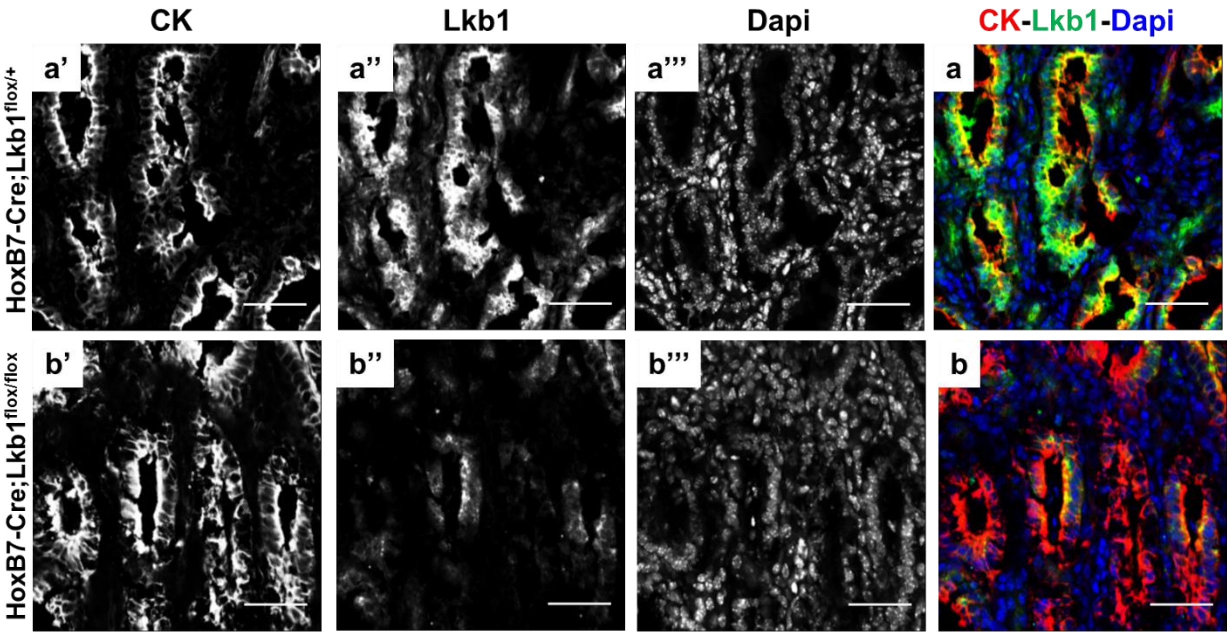

Supplementary Figure 1: Hoxb7-Cre efficiently deletes Lkb1

Sections through HoxB7-Cre; Lkb1$^{flox/+}$(a) or HoxB7-Cre; Lkb1$^{flox/flox}$ (b) P1 kidneys stained with antibodies to cytokeratin (red), Lkb1 (green) and the nuclear marker DAPI (blue). Individual channels for cytokeratin are in a' and b', Lkb1 a'' and b'' and Dapi in a''' and b'''. Scale bars equal 50 microns.

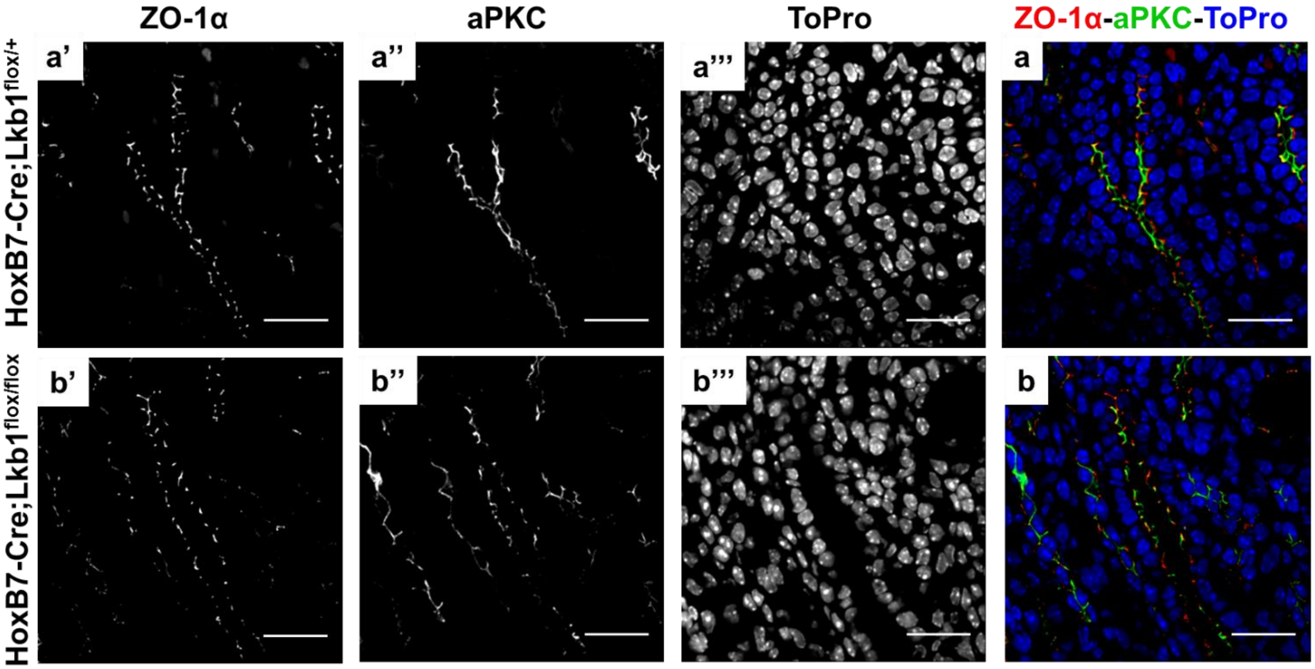

Supplementary Figure 2: Cell polarity is unaffected in Lkb1 mutant collecting ducts.

Sections through HoxB7-Cre; Lkb1$^{flox/+}$(a) or HoxB7-Cre; Lkb1$^{flox/flox}$ (b) P1 kidneys stained with antibodies to ZO-1α (red), aPKC (green) and the nuclear marker DAPI (blue). Individual channels for ZO-1α are in a' and b', aPKC a'' and b'' and DAPI in a''' and b'''. Scale bars equal 50 microns.

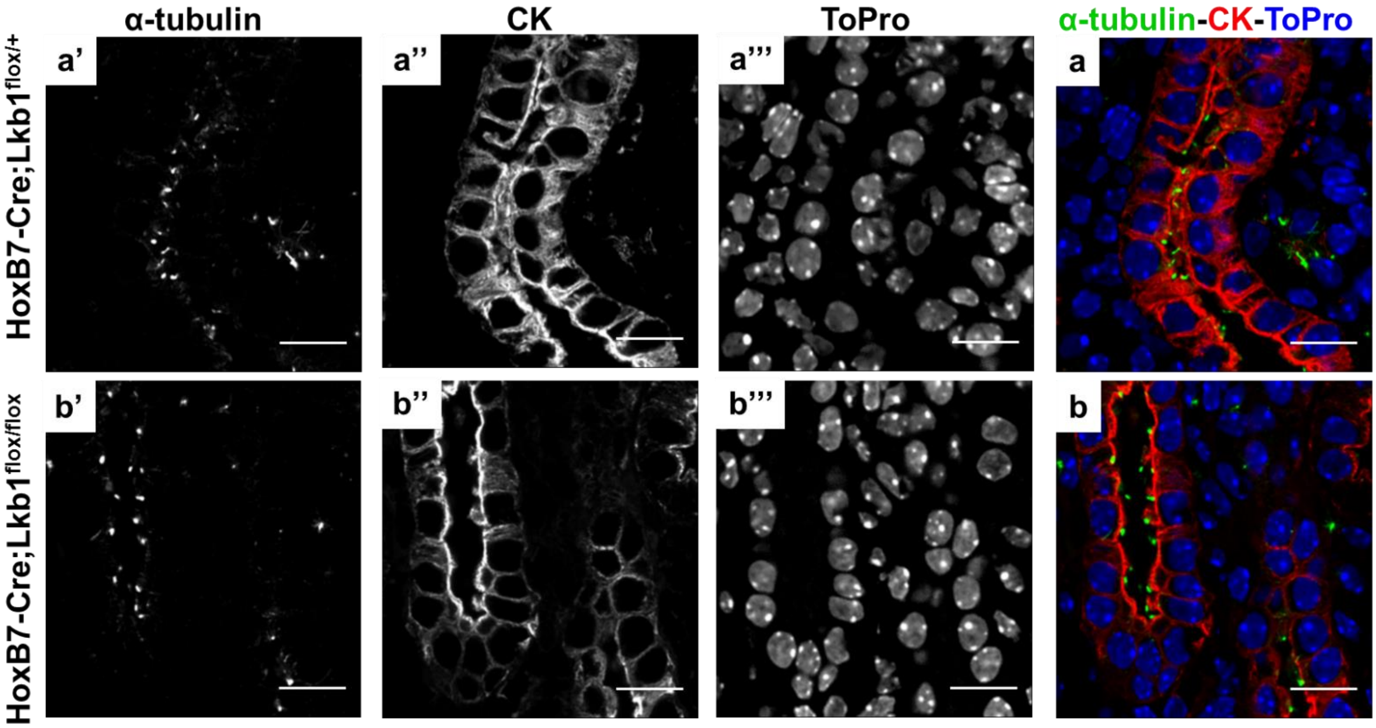

Supplementary Figure 3: Deletion of Lkb1 from the collecting ducts does not result in loss of cilia

Sections through HoxB7-Cre; Lkb1$^{flox/+}$(a) or HoxB7-Cre; Lkb1$^{flox/flox}$ (b) P1 kidneys stained with antibodies to cytokeratin (red), acetylated α-tubulin (green) and the nuclear marker ToPRO (blue). Individual channels for cytokeratin are in a' and b', α-tubulin a'' and b'' and ToPRO in a''' and b'''. Scale bars equal 5 microns.

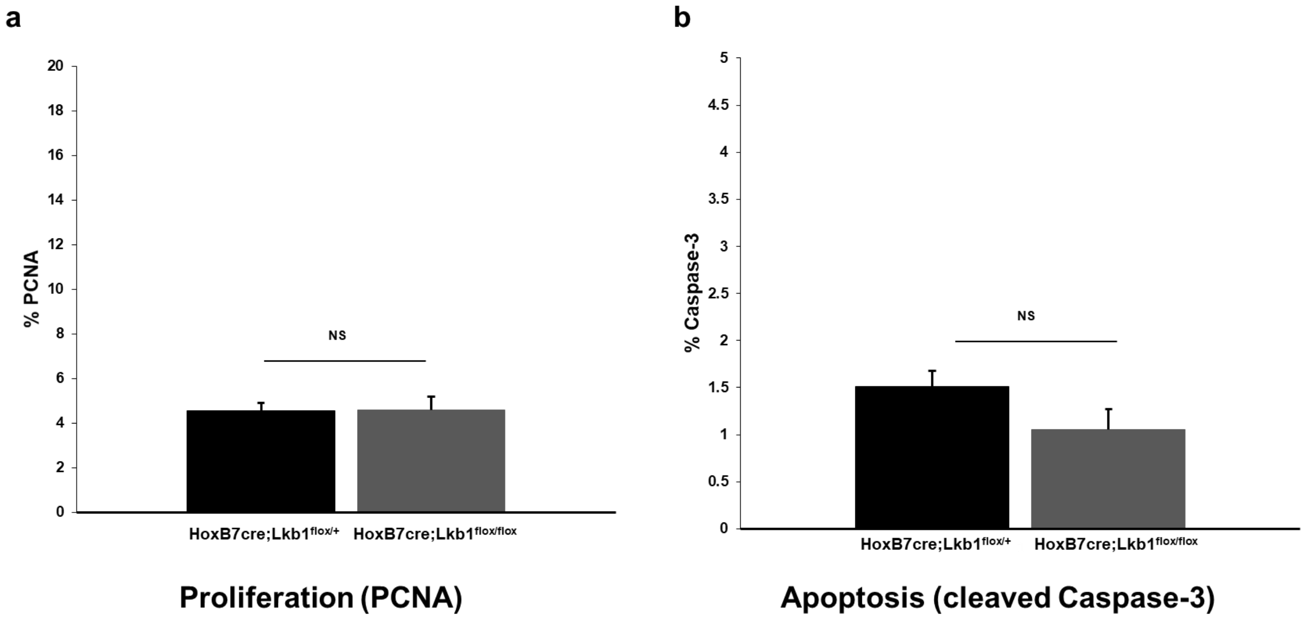

Supplementary Figure 4: Deletion of Lkb1 does not affect proliferation or apoptosis within the collecting ducts

(a) Quantification of cell proliferation via proliferation cell nuclear antigen (PCNA) within the collecting ducts of P1 HoxB7-Cre; Lkb1$^{flox/+}$ (n=5) and HoxB7-Cre; Lkb1$^{flox/flox}$ (n=5) sectioned kidneys. (b) Quantification of cell death via active caspase-3 within the collecting ducts of P1 HoxB7-Cre; Lkb1$^{flox/+}$ and HoxB7-Cre; Lkb1$^{flox/flox}$ sectioned kidneys. Statistical analysis via Mann-Whitney U-test. NS, not significant. Error bars shown as mean +/- standard error of the mean (SEM).

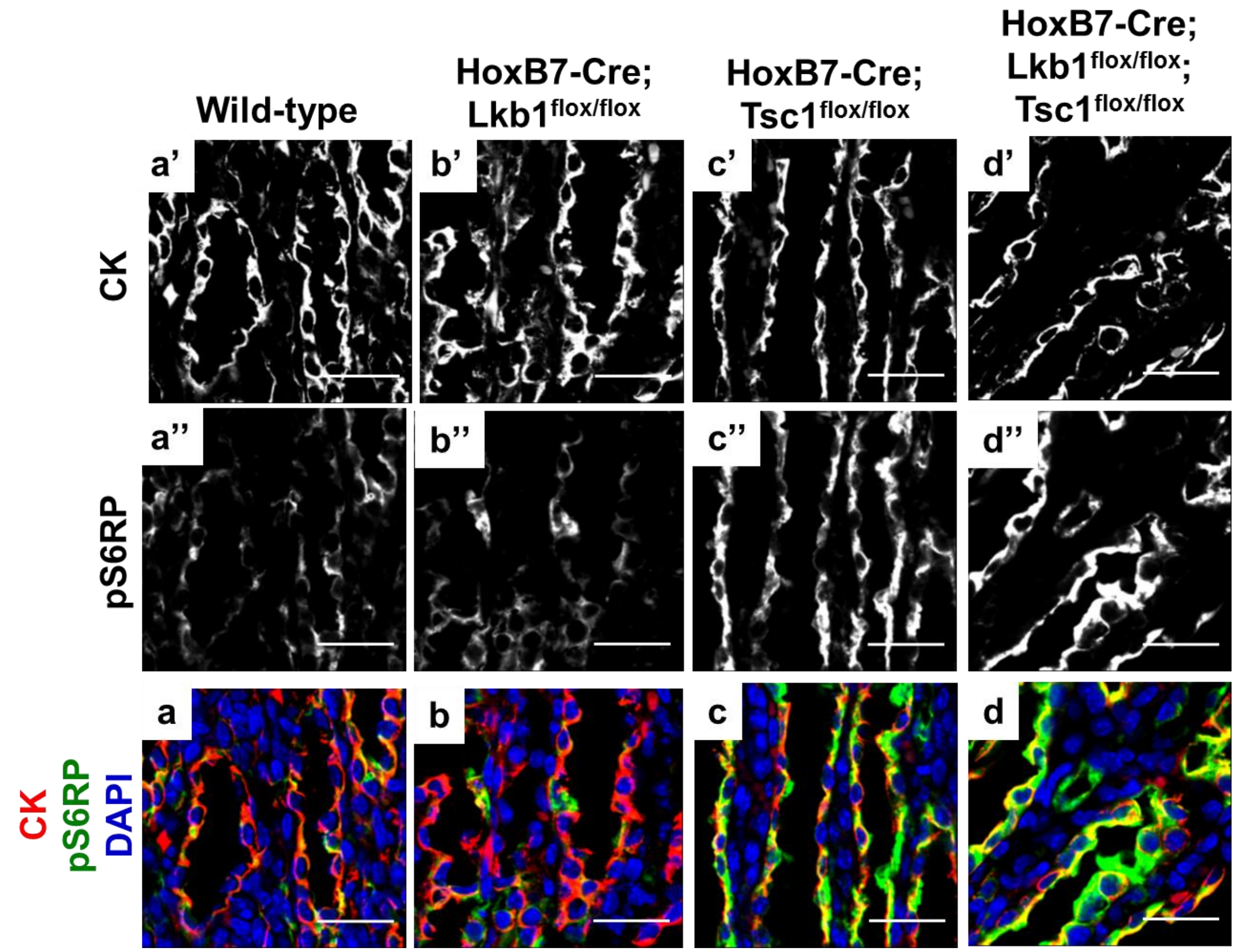

Supplementary Figure 5: Lkb1 mutant collecting ducts show no signs of mTOR pathway activation.

Sections through HoxB7-Cre; Lkb1$^{flox/+}$; Tsc1$^{flox/+}$ (n=5) (a), HoxB7-Cre; Lkb1$^{flox/flox}$ (n=5) (b), HoxB7-Cre; Tsc1$^{flox/flox}$ (n=5) (c), and HoxB7-Cre; Lkb1$^{flox/flox}$; Tsc1$^{flox/flox}$ (n=5) (d) P7 kidneys stained with antibodies to cytokeratin (red), phospho-S6RP (green), and the nuclear marker DAPI (blue). Individual channels for cytokeratin are in a', b', c', and d'; phospho-S6RP a'', b'', c'', and d''. Scale bars equal 50 microns.

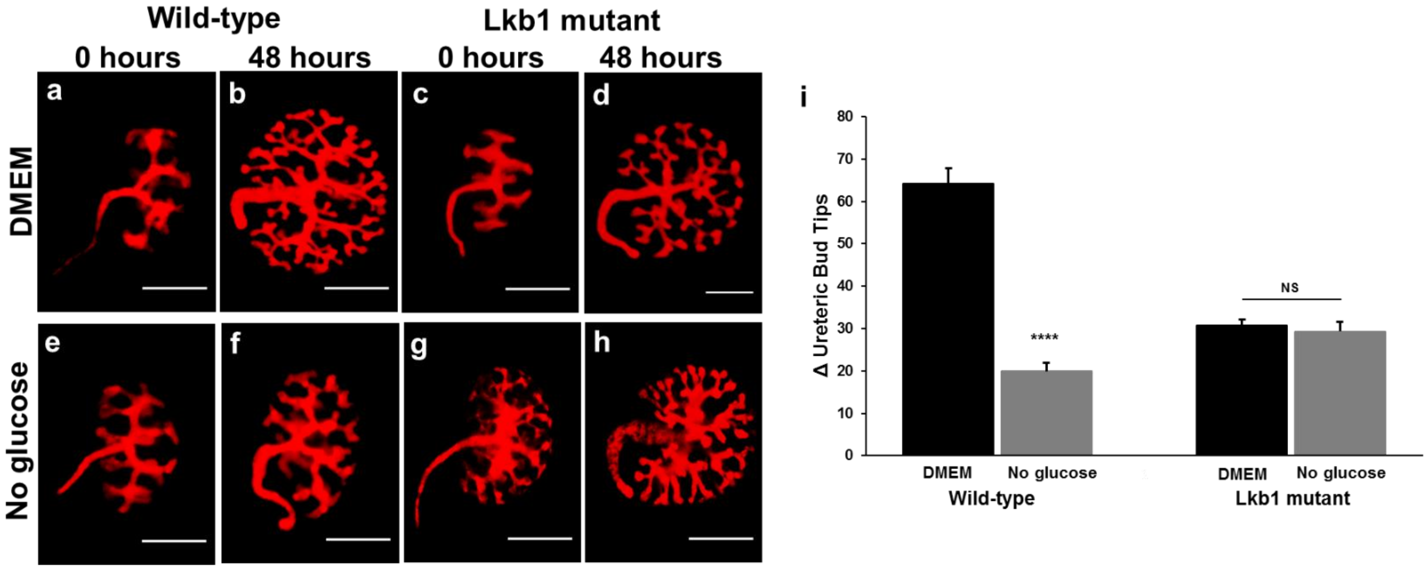

Supplementary Figure 6: Lack of glucose does not alter collecting duct growth in cultured Lkb1 mutant kidneys

Live images of E12.5 HoxB7-Cre; RosaTomato (n=10) (a,b,e,f) and HoxB7-Cre; Lkb1$^{flox/flox}$; RosaTomato (n=10) (c,d,g,h) kidneys after 0 (a,e,c,g) or 48 (b,f,d,h) hours of culture in control media (DMEM, a-d) or DMEM lacking glucose (e-h). Quantification of the change (Δ) in branch number for wild-type and Lkb1 mutant collecting ducts (i). n=10 for each genotype under each condition. Statistical analysis via Mann-Whitney U-test.****$p < 0.0001$, NS, not significant. Error bars shown as mean +/- standard error of the mean (SEM). Scale bars equal 30 microns.

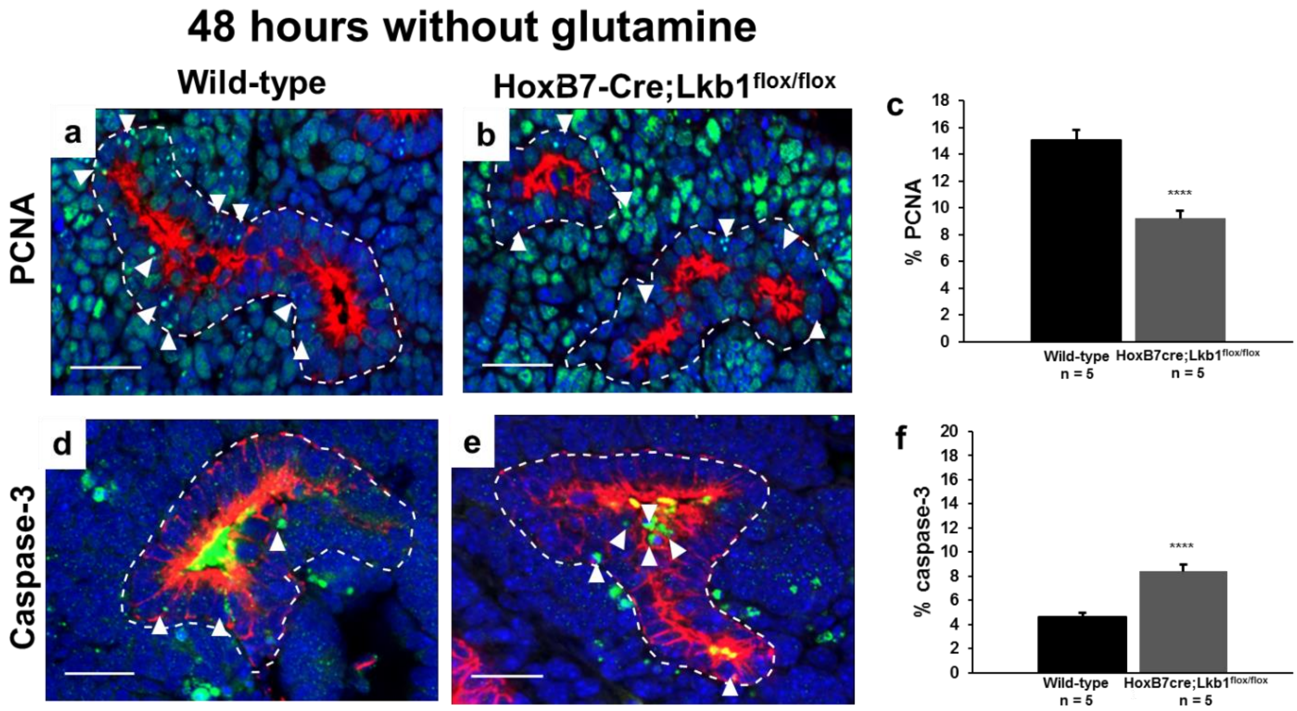

Supplementary Figure 7: Glutamine withdrawal causes decrease in proliferation and increase in apoptosis in Lkb1 mutant kidneys

Sections through E12.5 Hoxb7-Cre; Lkb1$^{flox/+}$ (a,d) and Hoxb7-Cre; Lkb1$^{flox/flox}$ kidneys after 48 hours of culture in media lacking glutamine. Sections were stained with PCNA (green in a and b) or an antibody to capase-3 (green in d and e) and the collecting duct marker cytokeratin (CK, red) and DAPI (blue). The percentage of PCNA positive or Caspase-3 positive collecting duct cells was determined for 5 distinct kidneys and the average is presented in c and d, respectively. Statistical analysis via Mann-Whitney U-test. ****$p < 0.0001$. Error bars shown as mean +/- standard error of the mean (SEM). Scale bars equal 20 microns.

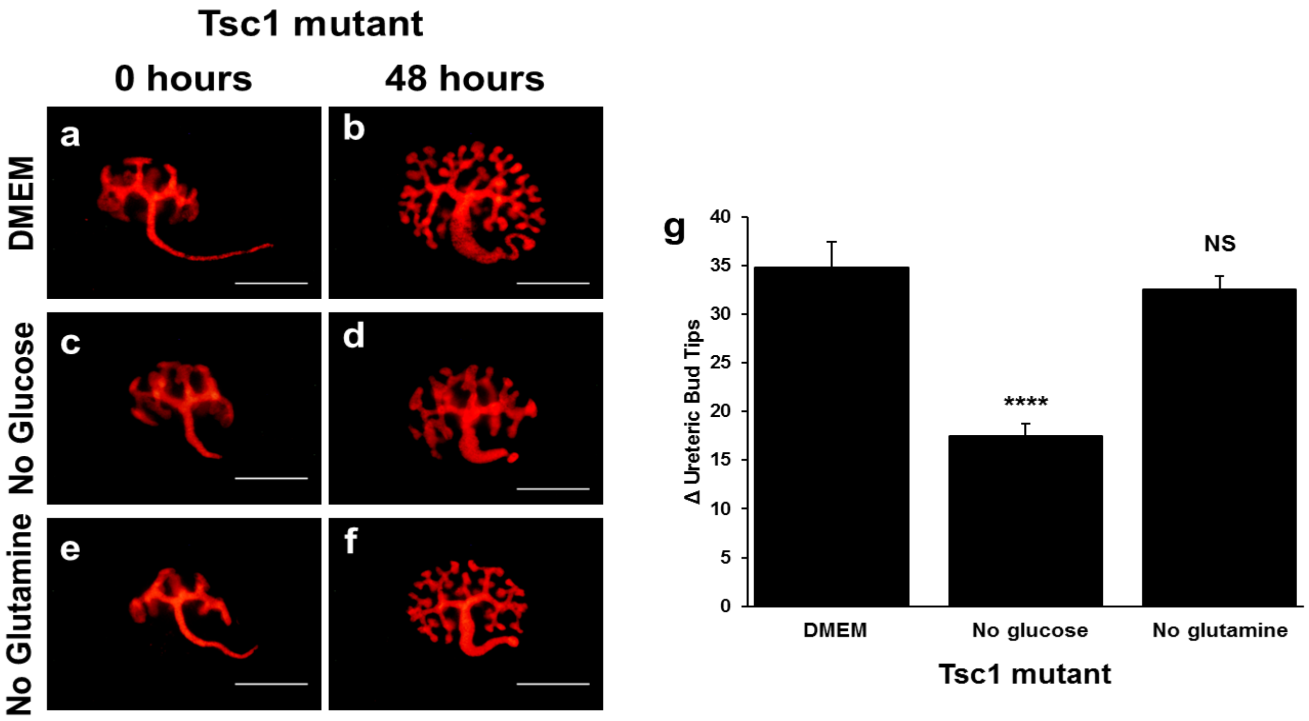

Supplementary Figure 8: Absence of glutamine does not alter collecting duct growth in cultured Tsc1 mutant kidneys

Live images of E12.5 HoxB7-Cre; Tsc1$^{flox/flox}$; RosaTomato kidneys after 0 (a,c,e) or 48 (b,d,f) hours of culture in complete media (DMEM, a,b), glucose deficient media (c,d) or glutamine deficient media (e,f). Quantification of the change (Δ) in branch number for Tsc1 mutant collecting ducts (g). n=10 for each condition. Statistical analysis via Mann-Whitney U-test. ****p < 0.0001, NS, not significant. Error bars shown as mean +/- standard error of the mean (SEM). Scale bars equal 30 microns.

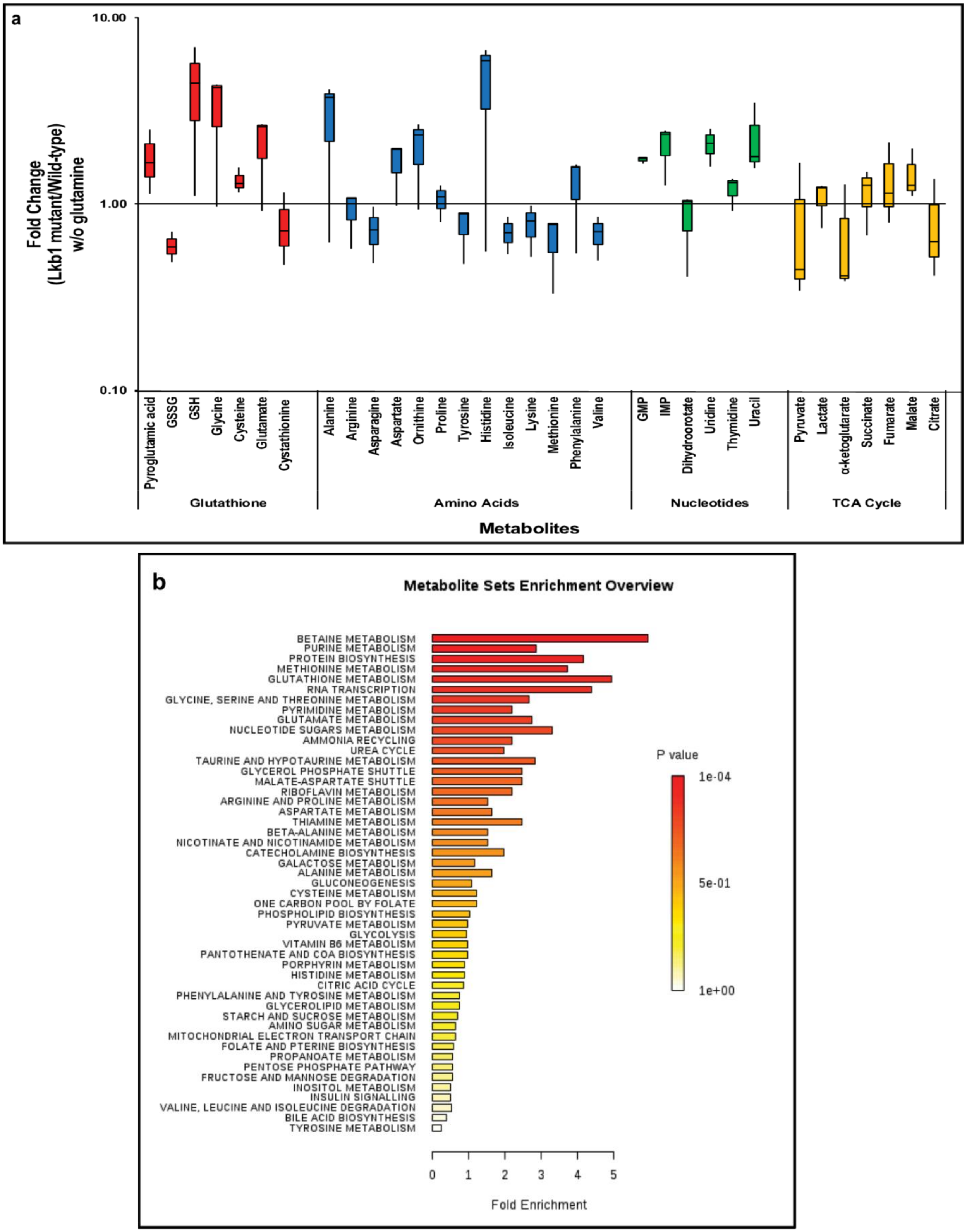

Supplementary Figure 9: Metabolomics Enrichment Analysis

Box plot of fold changes of key metabolites in glutathione, amino acid, nucleotide, and TCA cycle metabolic pathways during glutamine withdrawal (a). Variable importance (VIP) scores were

calculated and compared between wild-type and Lkb1 kidneys under control and glutamine-free conditions. Metabolites with a VIP score over 1.00 were considered to be significant in determining the classes used for metabolite set enrichment analysis (MSEA) (b). Metabolomics was performed on 3 independent samples containing 20 intact e13.5 kidneys per sample cultured in the presence and absence of glutamine. Statistical analysis via student's t-test. Error bars shown as mean +/- standard error of the mean (SEM).

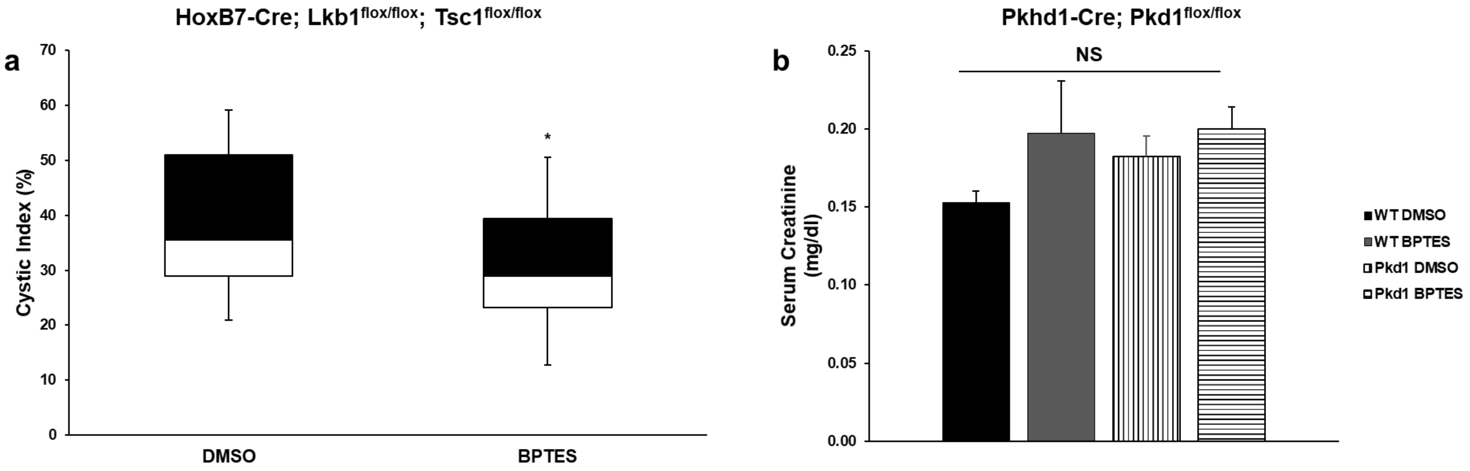

Supplementary Figure 10: Glutaminase inhibitor, BPTES, has no effect on serum creatinine of Pkd1 mutant kidneys

Comparison of cystic indices for DMSO vs BPTES-treated HoxB7-Cre; Lkb1$^{flox/flox}$; Tsc1$^{flox/flox}$ kidneys shows a significant reduction in the number of cysts in BPTES-treated animals (a). Quantification of serum creatinine of wild-type and Pkhd1-Cre; Pkd1$^{flox/flox}$ kidneys treated with DMSO or the glutaminase inhibitor, BPTES (b). Statistical analysis via Mann-Whitney U-test. *$p < 0.05$, NS, not significant. Error bars shown as mean +/- standard error of the mean (SEM).

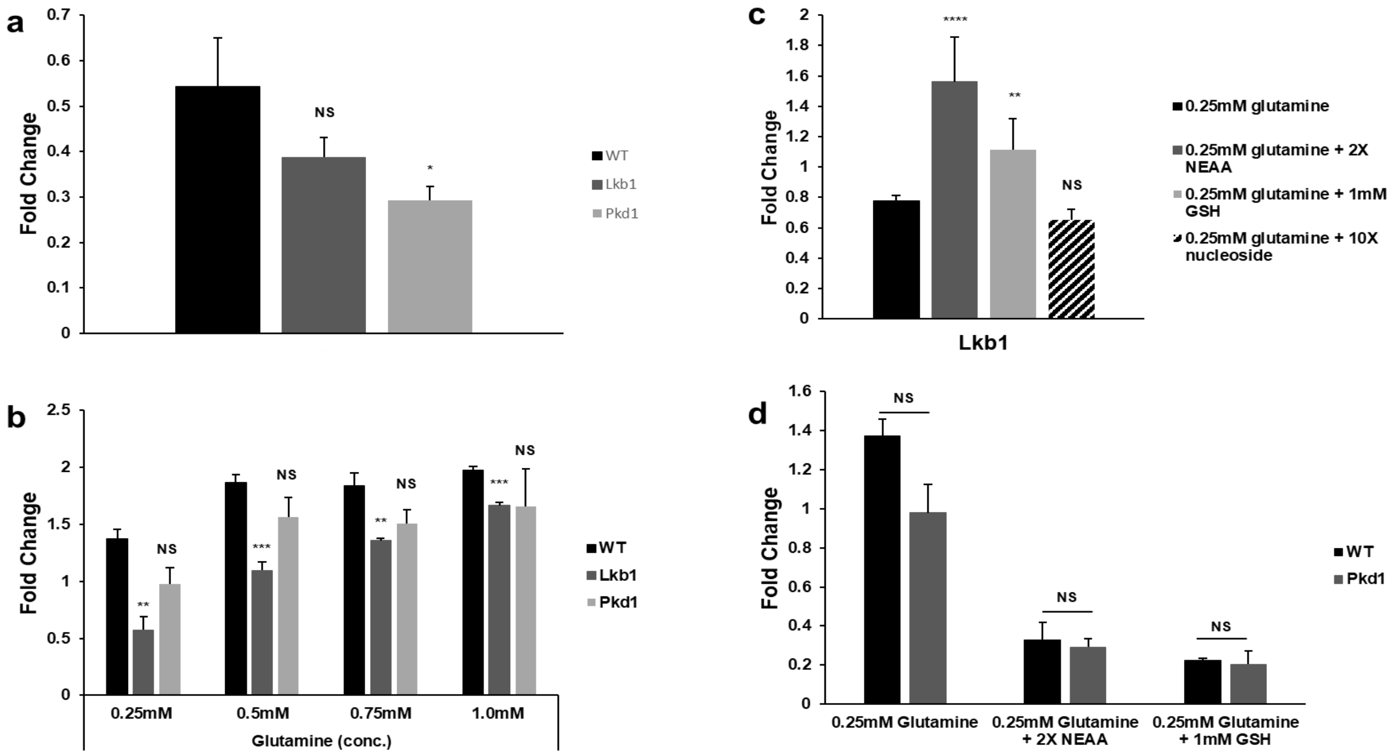

Supplementary Figure 11: Decreases in glutamine concentration causes decrease in proliferation in isolated Lkb1 mutant collecting duct cells

Quantification of fold change in growth from T=0 to T=48 hours in DMEM lacking glutamine (a). Quantification of fold change in specified (0.25mM-1.0mM) glutamine concentration in control (WT), Lkb1 mutant, and Pkd1 mutant inner medullary collecting duct (IMCD) cells (b). Quantification of fold change of Lkb1 mutant (c) and Pkd1 mutant (d) IMCD cells in low glutamine (0.25mM), low glutamine with non-essential amino acid mix (without glutamine), low glutamine with 1mM reduced glutathione (GSH), and low glutamine with nucleoside mix. Statistical analysis via student's t-test. *p <0.05, **p < 0.01, ***p < 0.001, ****p < 0.0001, NS, not significant. Error bars shown as mean +/- standard error of the mean (SEM).

Supplementary Table 1

Primer sequences for PCR genotyping are as follows:

HoxB7-Cre	5’-GGTCACGTGGTCAGAAGAGG-3’
	5’-CTCATCACTCGTTGCATCGA-3’
Pkhd1-Cre	5’-AGGTTCGTTCACTCATGG-3’
	5’-TCGACCAGTTTAGTTACC-3’
RosaTomato	5’-AAGGGAGCTGCAGTGGAGTA-3’
	5’-CCGAAAATCTGTGGGAAGTC-3’
	5’-GGCATTAAAGCAGCGTATCC-3’
	5’-CTGTTCCTGTACGGCATGG-3’
Lkb1	5'-GGGCTTCCACCTGGTGCCAGCCTGT-3'
Pkd1	5’-CCGCTGTGTCTCAGTGTCTG-3’
	5’-CAAGAGGGCTTTTCTTGCTG-3’
Tsc1	5’-AGGAGGCCTCTTCTGCTACC-3’
	5’-CAGCTCCGACCATGAAGTG-3’
	5’-AGCCGGCTAACGTTAACAAC-3’
