# Supplementary material for: Lkb1 deficiency confers glutamine dependency in polycystic kidney disease
Source: Nat Commun. 2018 Feb 26;9:814. doi: 10.1038/s41467-018-03036-y (PMC5827653; doi:10.1038/s41467-018-03036-y)
Supplement: Supplementary file 2 — Peer Review File [file 41467_2018_3036_MOESM2_ESM.pdf]

Editorial Note: this manuscript has been previously reviewed at another journal that is not operating a transparent peer review scheme. This document only contains reviewer comments and rebuttal letters for versions considered at Nature Communications.

REVIEWERS' COMMENTS:

Reviewer #1 (Remarks to the Author):

The authors have done an adequate job of addressing and responding to the reviewer comments from the initial review. The biology described in the paper is of broad interest and the data are of high quality.
